# The Co-Operative Holiday System

**Published:** 1915-07-31

**Authors:** 


### THE CO-OPERATIVE HOLIDAY SYSTEM.

Among the grievances of general practitioners, none is probably regarded as more irksome than the circumstance that he is never really off duty; week-days, Sundays, and holidays provide no security in this respect, though the amount of work performed on Sundays is usually less than that done on the other days of the week. The shortest absence requires that someone shall be told when he is due back, or where he may be found. Rumours of mutual arrangements between the practitioners of a compact district have from time to time been commented upon in the medical journals, but professional jealousy and local considerations have prevented much real progress in this direction.

The war has certainly had an influence on this matter. In fact it has had two opposing influences. On the one hand, the withdrawal of many practitioners from their civilian work in the early days of the war, and the extra work thrown thereby upon their remaining colleagues, and on account of the rashly volunteered service for the free treatment of dependants of soldiers, make some arrangement for obtaining a little rest absolutely necessary. Later on the further depletion in several districts of the supply of medical men made it, on the other hand, exceedingly difficult to formulate any plan by which each one of the group of doctors could have a day off, or at least half a day's rest, in rotation. Nevertheless, here and there it has been found possible to arrange that only one of a small group of doctors living in the same locality shall be found at home on a Sunday afternoon, or on a certain evening in the week. The desirability of such a break in the strain that is now the portion of any practitioner who is earning an adequate income requires no emphasis. A definite arrangement is obviously very much fairer than a haphazard early closing of the surgery, and "chancing the calls." Naturally the question of fees for work done is also the subject of a mutual agreement.

Owing to the enhanced fees now payable to *locum tenentes* and the difficulty of obtaining these—a difficulty which, we are informed, is rather exaggerated at the present moment—groups of practitioners in certain districts have elaborated a system by which the work of each is carried on during his absence on a few weeks' holiday. On this system, the temporary absentee relies on his remaining colleagues collectively, and not wholly on the nearest or the most friendly. Cards are made ready which show the names of the doctors willing to act for the "holiday-maker," and such a card is given to the patient, or to the messenger who calls at the absent doctor's house. The doctor eventually called in suitably endorses the card, and attends the patient until the return of the colleague from his holiday. Then the cards are collected and accounts "squared" on some approved basis. It is probable that when normal times return such co-operative measures may be continued and widely imitated, as they seem capable of being successfully extended. If this possibility be realised after the war, the professional *locum tenens* may find the demand for his services materially diminished in the future.

